# Medical student perspectives on substance misuse education in the medical undergraduate programme: a grounded theory approach

**DOI:** 10.1186/s12909-023-04145-z

**Published:** 2023-04-02

**Authors:** Senthan Rudrakumar, Natasha Varshney, Reverend David Taylor

**Affiliations:** 1 University of Liverpool, Liverpool, UK; 2 Gulf Medical University, Ajman, United Arab Emirates

**Keywords:** Substance misuse education, Addiction in medical school, Informal learning, Student voice, Curriculum change

## Abstract

**Background:**

Substance misuse teaching within the undergraduate medical curriculum has been underrepresented compared to more traditional medical topics. In response several national curriculum reviews such as the most recent UK department of health initiative (DOH) have identified deficiencies in substance misuse education and have suggested curriculum interventions for local faculties to implement. The student perspective however has largely been muted during this process and this study aims to explore this using a constructivist grounded theory approach.

**Methods:**

Eleven final year and intercalating medical students across three separate focus groups participated in this study over a three-month period commencing from March 2018. Time between the audio recorded focus groups allowed for a parallel process of data collection and analysis into more focussed codes and categories to occur, consistent with the grounded theory approach. The qualitative study took place in a single medical school in the UK.

**Results:**

Medical students had a common consensus that substance misuse education was an underperforming subject in their curriculum, from limited teaching hours to curriculum design and organisational problems. Students identified an alternative curriculum is required to not only prepare students for their future clinical duties but also their own personal lives. Students highlighted this proximity to a ‘dangerous world’ where exposure to substance misuse risks were faced daily. This exposure also provided a source of informal learning experiences which students deemed as being potentially unbalanced and even dangerous. Students also identified unique barriers to curriculum change with reference to a lack of openness due to the impacts of disclosure in substance misuse.

**Conclusion:**

Large scale curriculum initiatives appear to correspond to the student voice obtained in this study, providing backing for the implementation of a co-ordinated substance misuse curriculum within medical schools. The student voice however provides an alternative lens by outlining how substance misuse pervades into students’ lives and how informal learning is a largely underestimated hidden source of learning with more dangers than benefits. This together with the identification of further barriers to curriculum change, provide space for medical faculties to incorporate and work with students to facilitate local level curriculum changes relating to substance misuse education.

**Supplementary Information:**

The online version contains supplementary material available at 10.1186/s12909-023-04145-z.

## Introduction

Substance misuse and drug addiction levels are increasing. The devastating impact on mental health and society have been compounded by the COVID-19 pandemic due to increased social isolation and restrictions placed on treatment centres. Medical professionals who are often treating patients with substance misuse disorders are not isolated from this societal problem. Rates of substance misuse amongst the medical profession and student population have been reported to be disproportionately high and have often been linked with coping strategies for work related stress and pressures [1, 2].

Health policy has reflected a progressive attitude towards addiction with greater acceptance and understanding of the prevalence of such issues and management approaches focussing on rehabilitation and integration back into society. The complexity of this problem however is highlighted in the government’s latest 2021 announcement of a ‘10-year Drug Strategy’ which aims to tackle drugs and prevent crime, backed by the largest ever increase in funding for drug treatment [3].

With government and health strategies reflecting this growing recognition of substance misuse issues, it brings the question of whether medical education is responding appropriately to the pervasive subject of substance misuse.

Medical education in the UK began with a policy shift by incorporating substance misuse within the General Medical Council (GMC) Tomorrow’s Doctor Outcomes [4]; which outline the outcomes to be incorporated into all UK medical schools. GMC outcome 1.9 g states that graduates should be able to; *‘Identify and appropriate strategies for managing patients with dependence issues and other demonstrations of self-harm).’*

A UK Government department of health (DOH) funded project initiated in 2005 aims to implement a co-ordinated substance misuse curriculum into all UK medical schools [5]. The initiative consisted of three phases: Phase 1 (2005–2007) involved evaluation of current substance misuse teaching practices and deficiencies. The findings of phase 1 gave rise to a landmark substance misuse curriculum that outlined a uniform approach to teaching of substance misuse, orientated around six core learning topics (Table 1) which are further broken down into more specific learning objectives. Phase 2 (2008–2011) consisted of appointing curriculum champions to each of the 32 UK medical schools for implementation to proceed at a local level. Phase 3 (2013 –ongoing) looked towards sustaining those positive changes. The project concluded that training and education of student doctors had been enhanced and a solid basis for substance misuse teaching had been formed. The project stated that it had influenced the teaching and learning of at least 47,000 future doctors with benefits continuing to accumulate over time [6].

**Table 1:** Six core learning topics from DOH funded project

**1.** Bio-psycho-social models of addiction
**2.** Professionalism, fitness to practice and students own health
**3.** Clinical assessment of patients
**4.** Treatment interventions
**5.** Epidemiology, public safety, and society
**6.** Specific disease and specialty topics

Despite the landmark DOH curriculum project described above and a recent scoping review which highlighted that increasing educational interventions in substance misuse education were having a positive impact on medical students’ knowledge and attitudes [7]; local level translation to substance misuse rates within medical faculties has been limited. Recent anonymous surveys undertaken in 2021 in a single UK medical school highlighted that illicit substance use is now a recognised behaviour, with rates highest amongst the more senior year groups [8]. A further survey also captured how this interacted with mental well-being and possibly its effect on students’ fitness to practice [9].

Early researchers such as [10] who frequently re-visited the area of substance misuse education, pointed out that medical students should be part of the decisions and structures that go into improving the training for the next generation of trainees [10–12]. Inclusion of the student perspective can reduce resistance to change and thus enabling positive medical education reform [13].

The DOH curriculum however was constructed with the joint effort of a steering committee that was made up of predominately specialist clinicians including addiction specialists and psychiatrists as well as individuals with an interest in medical education. A key stakeholder group, possibly underrepresented in this were medical students themselves, a pattern that has been ongoing for some time. Faculty surveys carried out in 2004, highlighted a barrier to curriculum change in this field was that there were a high number of competing interests with the student perspective often muted [14].

A progress review of the DOH project carried out in 2014 highlighted that one of the key limitations was that the informal learning structures were unaccounted for [6]. The informal learning space is where the hidden curriculum is placed and is a derivative of the student perspective and voice [15]. A recent case report highlighted the importance of being aware of the hidden curriculum in substance misuse education, as the dangers and lethal impact the hidden curriculum resulted in one medical student having a relapse of their opioid use disorder during their training [16]. Further research highlights that by revealing this hidden sphere of learning, medical students can make more informed and empowered decisions, therefore influencing their own professional development [17].

By making the student the key driver for change, barriers toward implementation are much more likely to be overcome [14]. The top-down approach in the DOH curriculum project as well as prior research within this field misses a unique opportunity to explore student perspectives on substance misuse education, a health problem which is so different to many traditional curriculum topics because of its ubiquitous nature, which sees it potentially impacting both student’s personal and professional lives [2].

The objective of this study is to explore medical student’s perspectives on substance misuse teaching and learning within the undergraduate curriculum. This important viewpoint will aim to provide a useful lens in which to understand the subject area and provide useful information for policy makers, curriculum champions and medical educators when considering implementation or development of substance misuse education. The student perspective will also help to reveal aspects of the yet to be explored hidden curriculum referred to previously, which in turn may reduce areas of resistance when implementing large scale curriculum changes.

## Methods

### Design

The methodology adopted for the research question is based upon the qualitative approach of Constructive Grounded Theory by Charmaz [18]. Senior medical students were recruited via email invitation at serial timepoints between March and June of 2018, to allow for a series of focus groups to be undertaken. Ethical approval was provided by the University of Liverpool ethics board for a series of up to four focus groups, each with between 3–8 participants per focus group. Senior year groups were invited to participate as they were deemed to have higher rates of substance misuse as well as having had overall greater experience of their medical school curriculum than other cohorts to draw back from. E-mail invitations were sent out to 122 students of which 20 responded. 11 students participated in this study over three different focus groups. No further remaining students participated due to availability on the date of focus group. Further participants were not recruited as data sufficiency was deemed to be obtained, however with little known about the subject area and minimal theoretical sampling strategies adopted, theoretical saturation proved to be a challenge.

### Theoretical framework—grounded theory methodology

A qualitative approach is suitable to explore the student perspective of substance misuse education. Glaser and Strauss [19] developed the Grounded Theory approach during a time when qualitative analysis was seen as descriptive and less rigorous than quantitative research [20]. Glaser and Strauss [19] aimed to legitimise qualitative research by clarifying and codifying their procedures and practices for data analysis, enabling qualitative research to attain levels of rigour along-side those already well accepted quantitative methods [21]. The Grounded theory approach centres around the creation of theory via a parallel process of data collection and analysis which informs further points of data collection by the emergence of new concepts and theories. This inductive process eventually aims to develop theory from collected data [22, 23].

This approach is particularly suited to subjects where little is known, such as the exploration of medical student perspectives on substance misuse education.

Critiques of this approach however highlighted that the position of the researcher has potential to shape the emerging theory based on their own preconceived understanding of the subject area. The constructivist approach to grounded theory by Charmaz [18], however, allows for the researcher to use their understanding as a tool to greater understand the research participants by remaining open, yet facilitative in data collection. Constructive grounded theory considers the position of the researcher, their role and perspective, rather than the positivist notion that the researcher is a dispassionate and external analyst who is separate from the research field [23, 24].

### Researcher characteristics and reflexivity statement

The primary researcher and lead focus group facilitator (SR) for this study was a final year medical student, with an interest in medical education who undertook this study in 2018, as part of their intercalated master’s degree in Medical Education. The researcher subsequently went on to participate in further medical education postgraduate work. Furthermore, the researcher’s interest in substance misuse education was recognised following reflection of knowledge gained on the subject via exposure to the medical school curriculum, clinical attachments, and exposure to substance misuse in society. The researcher viewed their learning through the experiential learning lens, and they had the personal perspective that there was limited teaching on the subject in medical school in comparison to the level of exposure in society.

Adopting the constructivist notion that our backgrounds and viewpoints can determine our questions and interpretations of participants perspectives, the primary researcher worked closely with the project supervisor (DT) who took on a more neutral perspective on the research question. DT was a professor of medical education and had extensive experience in utilising the Grounded Theory approach. Emerging theory and concepts from data was frequently discussed in depth with DT and was documented in memo-writing form to guide further data collection. The combined discussion between SR and DT allowed for useful comparison of concepts and assumptions, thus providing a useful vantage point to which the student perspective can be observed and obtained until which point data sufficiency was discussed between SR and DT to have been adequately met.

Following completion of the data collection, analysis, and initial drafts, second author NV assisted in the editorial process to help prepare this manuscript for publication.

### Experiential learning

Experiential learning theory by Kolb [25], emphasises how knowledge is created through the transformation of experience. This learning theory was a lens through which the student perspective obtained in this study was viewed. The primary researcher SR had experienced the curriculum and had similar social interactions to the study participants and was therefore able to reflect on their own experiences to better capture the experiential learning being undertaken by study participants. A constructivist view on this methodology is that the knowledge is also determined by the social environment in which it is gained.

### Study population and context

The senior medical students who participated in this study as highlighted earlier (*n* = 11) were from a single UK medical school, spanning two-year groups of intercalating medical students (fifth year) and final year students (sixth year). The medical school in question at the time of the study being undertaken, consisted of a course set-up where the majority of all the curriculum was delivered prior to the fifth year and final summative examinations are sat in the fourth year of study. Final year medical students study consisted primarily of clinical attachment based in secondary care environments, whilst intercalating medical students were undertaking a higher study of research in their area of interest. Both year groups therefore had experienced most of their formal curriculum delivered by the medical school faculty. Thus, forming one of the key rationales behind the chosen study population. Details on demographics of study participants were not collected due to the sensitive nature of discussion as well as forming part of the ethical approval for this study.

### Focus groups

Focus groups provided a psychologically safe domain and suitable approach to data collection for a subject area that has the potential to be of a sensitive nature. Focus groups provide an opportunity to for participants to think broadly and discuss ideas within the group [18]. Focus group sessions were carried out by the primary researcher, who had experience and training in facilitating focus groups. The lead supervisor was also available for further guidance. Sessions were audio recorded with consent from participants. Sessions lasted between 50 to 90 min and took place in a pre-booked medical school provided conference room. The first two focus groups consisted of *n* = 4 participants each and the final focus group *n* = 3 participants.

Applying the constructive approach, the first focus group was centred around creating conversation. Charmaz [23] suggested initial data collection should facilitate discussion and openness using a flexible approach without frequent prompt or interviewing questions. Following analysis of the first focus group, additional guided questions were developed to explore emergent theory in subsequent focus groups until no new emergent categories were identified (see Table 2).

**Table 2:** Sample of focus group questions

*First focus group – open questions with further follow up questions*
*What do you all think about the teaching of substance misuse in medical school?* ­-*Can you recall any sessions you have had on substance misuse?* ­-*Were these sessions helpful?* *Why is it important to learn about substance misuse in medical school?* *-Have you seen patients who had substance misuse related problems?* ­-*Is it something you come across more often?* *Would you change the way substance misuse was taught in medical school, and if so how?* ­-*What kind of session would you create for medical students?* ­-*You mention its not as easy as creating a cardiology session but why?*
Second focus group—with guided questions in addition to 1^st^ focus group
*Has medical school prepared you sufficiently to manage patients with substance misuse problems?*
*Do you feel medical school can address substance misuse related problems in student’s personal lives?*
*If you were designing a new curriculum for medical students, how would you implement substance misuse education?*
*Do you feel there are any barriers in implementing this subject?*
Third focus group – further guided questions in addition to 2^nd^ focus group
*What other sources of learning apart from formal sources do you think students adopt regarding substance misuse education?*
*Does the following Model of substance misuse education relate to you?*
*What does being ‘streetwise’ mean to you?*

### Data coding and analysis

Audio recordings were transcribed after each focus group by the primary researcher using a word processing platform. Initial line-by-line coding commenced by attaching ‘gerunds’ as described by Charmaz [23] to ensure early codes remain objective and close to collected data (Appendix 1). This results in a large volume of fragmented codes obtained from continuous text.

A process of constant comparative analysis forms more focussed codes, where initial codes are refined and collapsed into larger codes (Appendix 2). Initial and focussed codes were also cross-checked by the research supervisor to ensure appropriateness of assigned codes.

Techniques such as Memo-writing and clustering were used to take ‘lower-level concepts’ to a higher level to provide more explanatory power and drive forward the emerging theory. Memo-writing is a note taking process as suggested by Charmaz [23] that provides space for questions to formulate about obtained codes (Appendix 3 and 4). Clustering is another technique which provides a non-linear, visual, and flexible method to understand and organise data. (Appendix 5). Clusters can outline the relationships of codes to each other and additionally highlight central codes, thus starting to produce tentative categories. Collaboration between the primary researcher and lead supervisor allowed for thoughts to be collected in memo-writing format, this was subsequently driven into clusters to highlight central core concepts.

### Data saturation

**Fig. 1:**
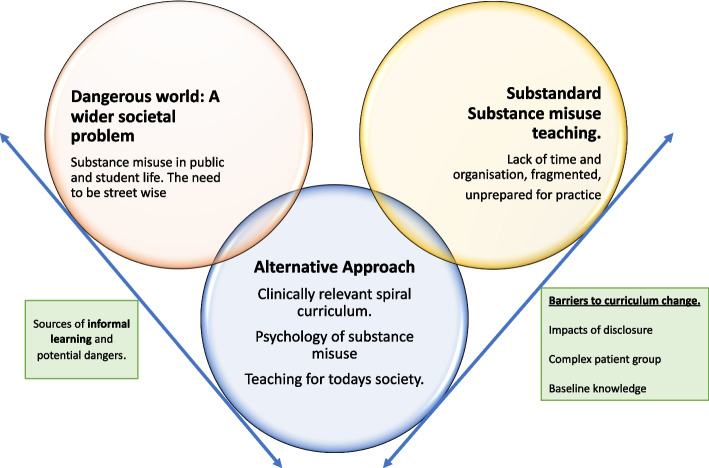
Suggested Model to represent Student voice on substance misuse education

The primary researcher and lead supervisor recognised that this study utilised a limited sample size, however following the third focus group, no further new core categories were identified. Further memo-writing and deliberation with the project supervisor, indicated that carrying out further focus groups would not create any further central core categories. A suggested model of the student perspective (Fig. 1) was presented and discussed with the third focus group participants (*n* = 3) and following this no further categories were also identified.

## Results

The framework suggested by Corbin and Strauss [26] using core and sub-categories is used to present the findings (Table 3), further to this a conceptual model was created to represent the interactions between these categories (Fig. 1). Three central core categories (1-3) and two further influencing core-categories (4-5) are identified and described below, supported by participant extracts.

**Table 3:** Core and subcategories of student perspectives

Core Category + *Influencing category*	Sub-Category
**1. Substandard substance misuse education: Unprepared to practice**	Curriculum design
	Tick box exercise
	Proportionality: importance vs time
**2. Dangerous world: A wider societal problem**	Substance misuse in public
	Substance misuse in student life
	Streetwise
**3. Alternative approach to substance misuse education**	Clinically relevant spiral curriculum
	Psychology of substance misuse
	Teaching for today’s society
**4. *Barriers to curriculum change***	*Complex patient group*
	*Impacts of disclosure: A need for openness*
	*Baseline knowledge*
**5. *Informal Learning***	*Sources of informal learning*
	*Dangers of informal learning*

### Substandard substance misuse education: unprepared to practice

Participants reflected on their learning experiences of substance misuse topics and recognised the deficiencies of the subject in their curriculum. Students frequently compared this to future roles as practicing clinicians and referred to a feeling of unpreparedness to practice in clinical situations involving substance misuse. This core category is further divided into the following: *Curriculum design, Tick box exercise* and* Proportionality.*

#### Curriculum design

Students reflected on individual teaching sessions involving substance misuse in their curriculum. Most students (*n* = 8) often struggled to recall any sessions at all, however following discussion and prompts by peers, students were able to draw on specific teaching sessions that they had experienced. Students also reported that sessions were fragmented, occurring sporadically within certain rotations such as the psychiatry rotation. Further to this, students reported that the timings of the sessions on substance misuse lends itself to poor attendance due to proximity to faculty examinations.

*“The only teaching we had was when we were on psychiatry in 4* ^*th*^ *year, and I remember one lecture in 1* ^*st*^ *year about substance misuse and professionalism issues. I think it should be planned better, it’s a matter of organisation.” (Participant 3).*

#### Tick box exercise

A key underlying reason frequently put forward for why teaching of substance misuse was limited was that the subject area was felt to be delivered in a tick-box manner to satisfy a third-party or national body requirement.

“I feel eLearning is now being used to tick off those taboo topics. It just helps with ticking a box for meeting a standard by the GMC.” (Participant 4)

“I feel eLearning is now being used to tick off those taboo topics. It just helps with ticking a box for meeting a standard by the GMC.” (Participant 3)

#### Proportionality

Teaching time dedicated to substance misuse within the curriculum was consistently compared to the volume of clinical exposure that participants felt that they would be exposed to as junior doctors. Participants were clear that there was a mismatch between the two. Students who were final year students who had completed their A&E rotations expressed this viewpoint more frequently than the intercalating students.

“Substance misuse is a highly prevalent problem in community but has the least amount of teaching dedicated to it. It’s something that in A&E you will face.” (Participant 8)

“When I was on my A&E placement, there was a lot of substance misuse related patients especially in the area surrounding my hospital. I feel university should prepare students for common things rather than rare conditions.” (Participant 7)

### Dangerous world: a wider societal problem

Substance misuse within wider society, separate to the formal curriculum environment made up a large proportion of the participant discussions and provided an insight into the dangerous world that medical students are exposed to. This world is explored via three key sub-categories: Substance misuse in public, substance misuse in student life and ‘Streetwise’.

#### Substance misuse in public

Students described their first-hand accounts and eye-witness reports of substance misuse taking place around them in everyday life. A key feature of the participants accounts included the prevalence and the proximity of the impacts of substance misuse to the students.

“I mean it’s everywhere, especially in big cities such as Liverpool. I see it every day when I come home from hospital, people using drugs outside my house. They do it in broad daylight!” (Participant 8)

“Yeah, it’s on our doorstep. It’s a dangerous world and we need to be better prepared.”(Participant 11)

“Substance misuse is a big problem here in Liverpool, I see so many patients in A&E who have addiction problems or needing treatment for withdrawal.” (Participant 5)

#### Substance misuse in student life

Substance misuse within the personal lives of students was a sensitive subject due to confidentiality reasons and possible concerns regarding fitness to practice. Despite this, students shed light on the impact of substance misuse within the medical student life and how help seeking is impacted by a lack of openness.

“Everyone forgets we are human beings; this could be an 18-year-old that has just come into university, we are told don’t do drugs, don’t do this. What happens when someone does do this? We are not told what to do” (Participant 6)

“I’m not going to lie, I heard about so many new drugs, like on nights out, people offer things to you and stuff. You just hear new random drugs that you’ve never heard of before.” (Participant 10)

#### ‘Streetwise’

Participants felt that substance misuse education can be dual purpose in not only preparing them for formal clinical duties but also as a responder to societal or personal demands Being ‘Streetwise’ was compared to being prepared for duties outside of the formal clinical environment in order to be able to respond to the above dangers of substance misuse within public and student life.

“I think we can be taught how to help a friend who is going through an addiction problem for example rather than just saying go and see a GP” (Participant 7)

“Being able to respond to someone who might have taken a drug on a night out and has reacted badly would be very useful” (Participant 11)

### Alternative approach to substance misuse education

Participants naturally outlined alternative approaches to substance misuse education having highlighted deficiencies with its delivery. The alternative approaches consisted of organisational design strategies as well as content.

#### Clinically relevant spiral curriculum

Participants felt that teaching should introduce basic theoretical underpinning principles in the early years of the curriculum. They also recognised the need to understand the clinical approach to substance misuse patients like that of other, more traditional subjects. Students suggested that this layered approach by introducing components into every year would lead to more sustained learning and a deeper understanding.

“In the first couple of years, you can get a decent grounding on the drugs which are common. When you get to 3^rd^ or 4^th^ or 5^th^ year, you can then plan. Some GPs will have addiction clinics, so you can spend the day there.” (Participant 3)

#### Psychology of substance misuse

Students discussed the need to understand the narrative behind substance misusers and those with addiction problems. Students felt that by listening to patient experiences with different substances and appreciating their journeys, students could understand the psychology underpinning patient choices.

*“It is going to be different for different people but in general there are certain triggers that lie within a certain spectrum, so to be able to see patients with these actual experiences, who have gone through this.” (Participant 5).*

#### Teaching to reflect today’s society

Substance misuse was viewed as a topic that was dynamic and ever evolving. Students felt the curriculum should reflect this by ensuring teaching was current and in line with today’s societal problems.

“The issue with current teaching is that it’s quite old school and didactic. You get some old professor teaching you about heroin; you get dilated pupils who get this … in reality, since substances are constantly changing it need to be constantly updated too: (Participant 11)

### Barriers to curriculum change

Specific barriers to curriculum change were identified within substance misuse education, and these can be categorised further into: Complex patient group, Impacts of disclosure and Baseline Knowledge

#### Complex patient group

Participants highlighted that when introducing more patient experience led sessions or addictions clinics, there was potential for unpredictability compared to other patient groups.

“It’s very hard for them to guarantee that they can sit you down in a group session with someone who has had a problem and tell you a story.”(Participant 7)

“So, it’s not as straight forward, as just getting people to come in. It’s a difficult topic. You are dealing with un-predictable people. Just thinking about how hard it can be to organise these things, it would be a credit to the medical school if they can organise that.” (Participant 9)

#### Impacts of disclosure: a need for openness

Students felt the culture of fear and lack of openness in substance misuse matters has the potential to limit curriculum change. Certain teaching environments such as group sessions may not be appropriate due to the risk of disclosures and subsequent professionalism concerns.

“Learning of each other, people will be a bit apprehensive because, everyone knows someone who takes drugs, and that person is not going to stand up in a session and discuss it. No one would want to be associated with that” (Participant 8)

“Maybe if there was openness, it would be easier for medical students, or all students to come forward and things will be stopped earlier rather getting to that point where there is no return. Or maybe an addiction happens.” (Participant 5)

#### Baseline knowledge

Another barrier which emerged was the prior understanding of the subject area being highly variable. Students were aware that they all had different backgrounds and upbringings, and this can impact on the learning of this subject. There was a consensus that education must cater for this with an aim to standardise knowledge.

“If you’re brought up in a certain environment, when its culturally taboo, you can’t really discuss it at home. So, it’s probably more of an issue. If you’ve come across it and you have never, and you don’t really know what it is and because you don’t really have that sort of experience, you may find it more difficult to deal with. In that respect, it is on the medical school to teach to an adequate standard.” (Participant 4)

“If you’ve gone to a school where it’s not talked about, or you don’t even see people in public doing it. Then your awareness will be less than a lay person, if it’s not brought to your mind that these things exist, you shouldn’t be shocked if you see someone in hospital who has taken 10 pills together. That’s one of the key issues, everyone comes in not knowing about what a stroke is, everyone starts from the same baseline and learns simple things. But every has different baseline understandings of it. People know different things, and everyone need to be bought up to some sort of similar standard.” (Participant 10)

### Informal learning

Informal learning refers to the learning away from the formal institutional structures. In this case away from the hospital or medical school setting. Two further categories emerged from this: Sources of informal learning and Dangers of informal learning.

#### Sources of informal learning

Students reported a wide variety of sources of settings of this informal learning. This usually emerged following the identification of limited formal sources of teaching on this subject.

“I used to watch a lot of these VICE videos on YouTube. They show how drugs are made and sold. They also look at the latest drugs that are becoming more popular and talk about their effects.” (Participant 10)

“It’s mainly from seeing people around us who may have gone through it, may know other people who have gone through it, like taken a drug, it ended badly, usually you hear the bad stories, so you hear, ah this happened to this person.” (Participant 2)

“I saw this TED talk online; this guy is quite a maverick and does what he wants. He used a presentation with only pictures, such us a picture of someone gurning on a drug, pictures of someone in a field. He used his own experience of when he lived in New Zealand. People sniff a lot of glue in New Zealand because it’s very rural. Not in New Zealand but in Bangladesh. It’s a good way to get high. He used anecdotal stories which were very interesting.” (Participant 4)

#### Dangers of informal learning

The informal learning sources identified highlighted an unbalanced view of substance misuse issues for students. Participants highlighted potential dangers of this method of learning for this subject.

“I think that is a also a dangerous game, because you get a lot of people that are kind of uneducated toward drugs, and have a different opinion. A lot of people, almost make it into a fashion.” (Participant 5)

“You hear a lot of people talk about drugs, and only mention the good parts. They tend to be the people who abuse drugs. Whereas if you get actual proper education on it, then you get the bad sides and the good sides. You never get the good sides, but you usually get the bad sides. If you usually hear it from a friend, that has taken some sort of drug, you usually hear the good parts of it. So, a lot of people can research about it one the internet, where you hear the good stuff, but you never hear the bad stuff. “(Participant 10)

## Discussion

### Summary of key findings

The suggested model (Fig. 1), which is formed through theory generation based on the constructivist grounded theory approach, aims to provide a visual framework of the interrelationships between the five categories that were coded for within the data derived from three focus groups. The student voice outlines specific aspects of an ‘alternative curriculum’ however the model suggested (Fig. 1) aims to highlight the importance of an alternative student-centred curriculum and its dual purpose in that it could not only respond to the sub-standard curriculum delivery (core-category 1) but is also able to appropriately respond to the dangers within society (core-category 2).

Despite this, potential barrier’s specific to substance misuse education were identified when considering curriculum change (core category 4). Barriers to curriculum change and Informal learning are seen as key findings that influence the ability to achieve an alternative curriculum and pose more of a rate limiting factor as highlighted in the suggested model of student perspectives (Fig. 1). The ‘informal learning’ (category 5) was a late emerging category that seemed to be initially isolated within the ‘Dangerous world’ (category 2). The Dangerous world exposed students to a hidden curriculum of learning, with the potential to expand this ‘Dangerous world’ without any formal context. Despite the close relationship of ‘Informal learning’ (category 5) and the ‘Dangerous world’ (category 4), informal learning was seen to influence student’s unpreparedness to practice, as well as themes within the alternative curriculum and even within student identified barriers to curriculum change. Due to its overarching nature, informal learning emerged as a separate category but with a highly influencing position together with barriers to curriculum change in the effectiveness of alternative approaches in substance misuse curriculum initiatives.

### Comparisons with existing literature

Substandard substance misuse curriculum delivery (Category 1) appears to be a recognised theme in existing literature with international calls for education and training in addiction medicine to be improved (category 3). Surveys concluded that teaching hours were very limited with some medical schools only having 3–6 h of dedicated teaching time in their programme [10, 11]. A survey of 98 medical schools from 1986–87 highlighted that teaching was restricted to a few specialties such as psychiatry. A further study identified that training in addiction medicine was half of that of other chronic disorders [27]. Much of the early literature on substance misuse education focussed on the quantifiable factor of number of teaching hours dedicated towards a particular subject. The student voice does reflect the sparsity of substance misuse teaching within the medical curriculum; however, it also makes clear that the quantifiable measure of teaching hours is only a single aspect and that other factors such as content of curriculum delivered, and student centeredness are equally important to consider.

The student perspective produced core category 2 – ‘A Dangerous world’, which goes beyond the formal curriculum parameters and explores substance misuse education from the perspective of its high prevalence within society and in students’ own lives. Current literature is largely from the perspective of faculty members who have undertaken curriculum reviews to understand the subject area. There is an increasing recognition of growing substance misuse issues amongst medical students, with recent anonymised surveys quantifying levels of substance misuse amongst individual faculty groups [7]. Further to this the UK DOH curriculum project has suggested learning objectives under the title ‘‘Professionalism, fitness to practice and students’ own health’ (Table 4) that appear to respond to the pervasiveness of this issue in student lives.

**Table 4:** Learning objects from DOH project which appreciate the 'Dangerous World'

Describe the risk factors for substance misuse in medical students and in health professionals
Describe how substance misuse problems may affect a health professional’s judgement, performance, and care of their patients
Describe the need to balance due concern for the health of a colleague with responsibilities for the safety and welfare of patients
Outline the role of the medical schools and the GMC in ensuring students’ and doctors’ fitness to practise

Describe the sources of help for students and doctors with drug and alcohol related problems

Despite this the learning outcomes (Table 4) adopt verbs such as describe and outline, which seem to settle for a lower level of learning and subsequent level of engagement. Instead, one learning outcome could be re-written according to Blooms Taxonomy 1956 [28], to ‘*Evaluate the current available sources of help for students and doctors with drug and alcohol related problems and how access and availability can be improved’.* This provides scope for and discussion amongst students and peers regarding current challenges in the availability of sources of help but also factors that determine someone accessing help. Assessment can take form of students possibly identifying or creating an alternative system for medical professionals to seek help to improve access and availability. This creates a move away from students learning in a didactic method on subjects that are particularly important to students personal and professional lives and re-visits the idea of not approaching crucial subjects in a tick-box exercise style as highlighted in the student voice.

With previous literature focusing on rates of substance misuse amongst medical students [29], the student voice provides a more explanatory lens by highlighting the close proximity of dangers and risks being faced in student lives. This voice emphasises the need to not only implement the above learning objectives (Table 4), but to ensure that appropriate stakeholders and systems are in place to ensure student wellbeing services are appropriate. For example, discussions around sources of help for students and doctors with drug and alcohol related problems can be tied in closely with the student wellbeing services, which in turn may lead to positive benefits to students as well as improving accessibility to student well-being services.

An alternative curriculum (core category 3) put forward by study participants addressed shortcomings in their own curriculum but also looked to address the ‘Dangerous world’ as visualised in the student voice model (Fig. 1). This provides some tentative signs of consistency between the student voice and the UK DOH curriculum put forward. The UK DOH curriculum initiative refers to co-ordinating the content, delivery, and assessment by ensuring core outcomes are covered at appropriate points making a ‘joined-up’ teaching approach for students. This will be facilitated by curriculum champions at faculty level with the aim of enabling long term consistent changes [5]. Considering the student voice on substance misuse education in general, it may seem intuitive to include senior students within the local level curriculum champion faculty discussions to ensure curriculum design and teaching sessions are appreciative of the ‘hidden curriculum’ and pervasiveness of the problem within students’ lives.

Barriers to curriculum change (influencing category 4) in substance misuse have previously been identified in existing literature, primarily from a ‘top-down’ approach from the perspectives of medical school deans, clinicians and curriculum leads. These barriers include the medical model of addiction not being appreciated, cuts on psychiatry placements, medical students not given priority and finally the potential emotional impact of substance misuse education on students with personal or family history of addiction problems [30]. The student voice identified further areas of resistance including the impacts of disclosure involved with substance misuse and students having differing prior knowledge and exposure of substance misuse, owing to the highly contextual and informal nature of the subject. The student identified barriers to change are closely tied with the sources and dangers of informal learning (category 5).

With an awareness of the exposure to substance misuse from the student perspective, informal learning (category 5) sources were increasingly apparent. Students understanding of substance misuse, a result of being exposed to this ‘dangerous world’, lent itself to be a major source of informal learning on the subject area. The DOH curriculum project makes clear that one of the study limitations was the exploration of informal learning sources that students are exposed to [5] Further to this current literature, on informal learning within this subject area is also very limited. The student perspective identified specific informal learning sources, and the consensus was that the informal learning often had a biased undertone and was deemed subjective based on the source of the informal learning. Students were aware that their knowledge had the potential to be unbalanced and potentially ‘Dangerous’. This is consistent with a recent unfortunate case analysis of a medical student who was a victim of opioid misuse, and the informal learning played a potential lethal role in the student’s journey [9].

### Strengths and limitations

The strengths of this study lie in its use of the constructivist grounded theory approach in generating a possible model to represent the student perspective in a growing area of interest within medical education. Substance misuse education within the medical curriculum has been evaluated from multiple different perspectives however the student voice has been a relatively muted on this subject. The inductive nature of the grounded theory approach has enabled subsequent areas of research to potentially be explored in further studies, for example the under explored ‘informal learning’ (category 5).

This study was however, carried out in a region of the UK with a particularly high rate of substance misuse amongst the general population [31] and some themes described by the study participants pertained directly to their medical school environment. Therefore, for a broader, more representative perspective this study could be repeated at different medical schools across the UK. Further to this despite data saturation requirements being met, the suggested model of student perspectives is based on a small study participant number (n = 11) of senior medical students, meaning it cannot be entirely representative of other year groups.

### Implications for educational practice

Despite the limitations described, this study provides a useful lens for individuals looking to implement local or specific national level changes regarding substance misuse education within the medical curriculum. The student perspective appears correspond with the current literature and curriculum reviews that make clear the lack of substance misuse teaching in the medical curriculum. The student perspective provides and alternate lens and relates the lack of teaching towards being unprepared for not only their future roles as a health practitioner but also as a member of society and as an individual. This duality is an important consideration when planning substance misuse teaching sessions as well as student well-being initiatives that can respond to substance misuse issues in the students’ personal lives. Faculty members and curriculum champions must also be aware of the presence of the informal learning taking place within this subject area. Implementing local level curriculum changes not only appreciates but challenge the knowledge gained through these informal sources. Revealing the informal social side to learning in this subject area as described by Hommes [32] can be crucial when designing individual teaching sessions which can provide an open space to utilise this sphere of learning.

Amid large-scale nationwide UK DOH curriculum initiatives, this relatively small-scale study at a single institution in the UK provides some early insight into the informal social context that, students appear to reflect and continuously draw from. With the absence of formal training, students are showing agency from drawing from informal life experience. Formalisation of the ‘hidden curriculum’ may take away from the spontaneity of the informal aspects of learning [32] however curriculum design can make use of this equally. One example could include an open discussion with an ex-substance misuse patient where students pre-conceived thoughts and ideas can be challenged and debated.

The student perspective revealed aspects of an alternative substance misuse curriculum which is largely in line with the UK DOH Curriculum initiative. This further emphasises the urgency and need to incorporate substance misuse education within the medical curriculum considering the rising prevalence of the problem in society. However, despite the recent curriculum initiatives and reported developments this has not necessarily led to local level changes as evident in the student voice obtained from a single institution. Curriculum champions and faculty members must therefore appreciate some of the areas of resistance as highlighted by the two influencing categories 4 and 5; ‘barriers to curriculum change’ and ‘informal learning’ to facilitate long term sustained changes within the medical curriculum.

### Implications for research

The inductive nature of the research study which revealed the under reported category of ‘informal learning’ within substance misuse education provides space for further research within this field. The use of focus groups allowed participants to interact with each other but also acted as a potential limitation when exploring the informal learning category. Focus groups remained open and undirected initially and thus providing space for free discussion. Participants being medical students could potentially be averse from discussing matters which may run the risk of any professionalism concerns being directed towards themselves. This not only re-iterates the barrier created by a lack of openness, but the sensitive and confidential nature required when continuing to further develop research initiatives in substance misuse within medical education. Recent studies have adopted to use anonymised questionnaires to explore substance misuse within medical students [8]. This however misses out on a potentially crucial opportunity to explore the student narrative on a subject that is so prevalent today.

The need for substance misuse education for health practitioners is being more widely recognised, with potential curriculums and programmes having been suggested [30]. Future research must continue to not only explore the perspectives of faculty members and education leads but also explore the student perspective following curriculum changes and incorporation of suggested teaching programmes. Future research must also look to explore the health care practitioner or students’ narrative on substance misuse issues and explore the ‘informal learning’ category identified in this small-scale study. This can potentially allow formal curriculums and medical education faculties be more responsive to students and therefore help prevent and manage substance misuse or addiction problems within medical students or health care practitioners.

## Conclusion

Substance misuse teaching within the undergraduate programme has by comparison been an increasingly underrepresented area of learning. This provided scope for research to examine methods to implement an alternative approach from the perspective of clinicians, faculty staff and curriculum organisers. The constructivist grounded theory approach explored the perspectives of medical students on substance misuse education and emphasised a central principle that substance misuse curriculum must not only focus on preparedness for practice but preparedness for the dangers of substance misuse within daily life. This theme aligned with other key stakeholder perspectives within the DOH backed curriculum project and therefore providing support for the implementation of DOH learning outcomes. The student voice however revealed two key areas of resistance, that are in themselves opportunities to enhance student engagement: the largely underestimated sources of informal learning, as well as barriers to curriculum change. With curriculum changes now being suggested at national levels and rising rates of substance misuse recognised in society and amongst medical students, medical faculties should consider firstly evaluating the student perspective on substance misuse education in their own institutions and incorporating this relatively muted voice in curriculum delivery and implementation at a local level.

## Supplementary Information

**Additional file 1:** **Appendix 1.** Section of initial coding (focus group 1). **Appendix 2.** Section of initial coding transformed into focussed coding (focus group 1). **Appendix 3.** Real life substance misuse knowledge memo. **Appendix 4.** Pre-conceived idea / thoughts memo. **Appendix 5.** Formation of future Careers cluster.

## Data Availability

The datasets generated and/or analysed during the current study are not publicly available due to participant confidentiality as per consent obtained from participants. However anonymised and restricted focus group transcripts are available from the corresponding author on reasonable request.

## Abbreviations

DOH: Department of health
UK: United Kingdom
