# Supplementary material for: Medical student perspectives on substance misuse education in the medical undergraduate programme: a grounded theory approach
Source: BMC Med Educ. 2023 Apr 2;23:205. doi: 10.1186/s12909-023-04145-z (PMC10068233; doi:10.1186/s12909-023-04145-z)
Supplement: Supplementary file 1 — Additional file 1: Appendix 1. Section of initial coding (focus group 1). Appendix 2. Section of initial coding transformed into focussed coding (focus group 1). Appendix 3. Real life substance misuse knowledge memo. Appendix 4. Pre-conceived idea / thoughts memo. Appendix 5. Formation of future Careers cluster. [file 12909_2023_4145_MOESM1_ESM.docx]

**Appendix**

**Appendix 1 Section of initial coding (focus group 1)**


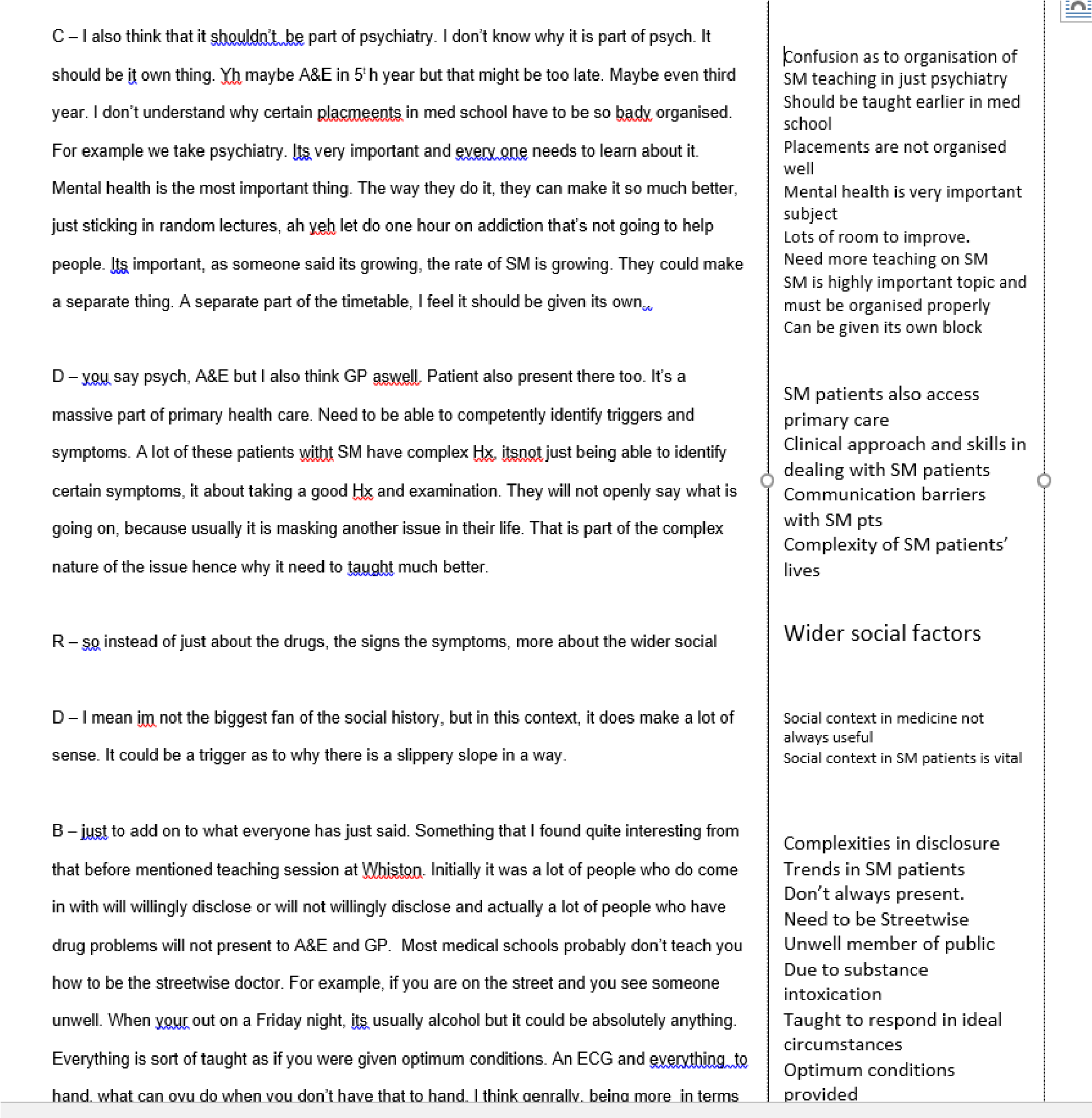


**Appendix 2 – Section of initial coding transformed into focussed coding (focus group 1)**


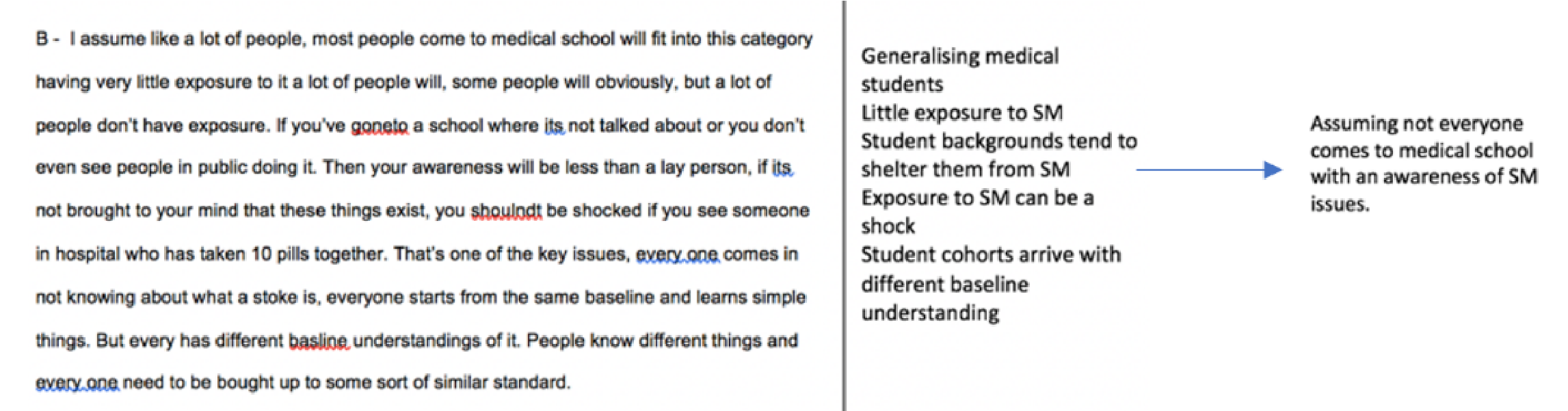


**Appendix 3 Real life substance misuse knowledge memo**


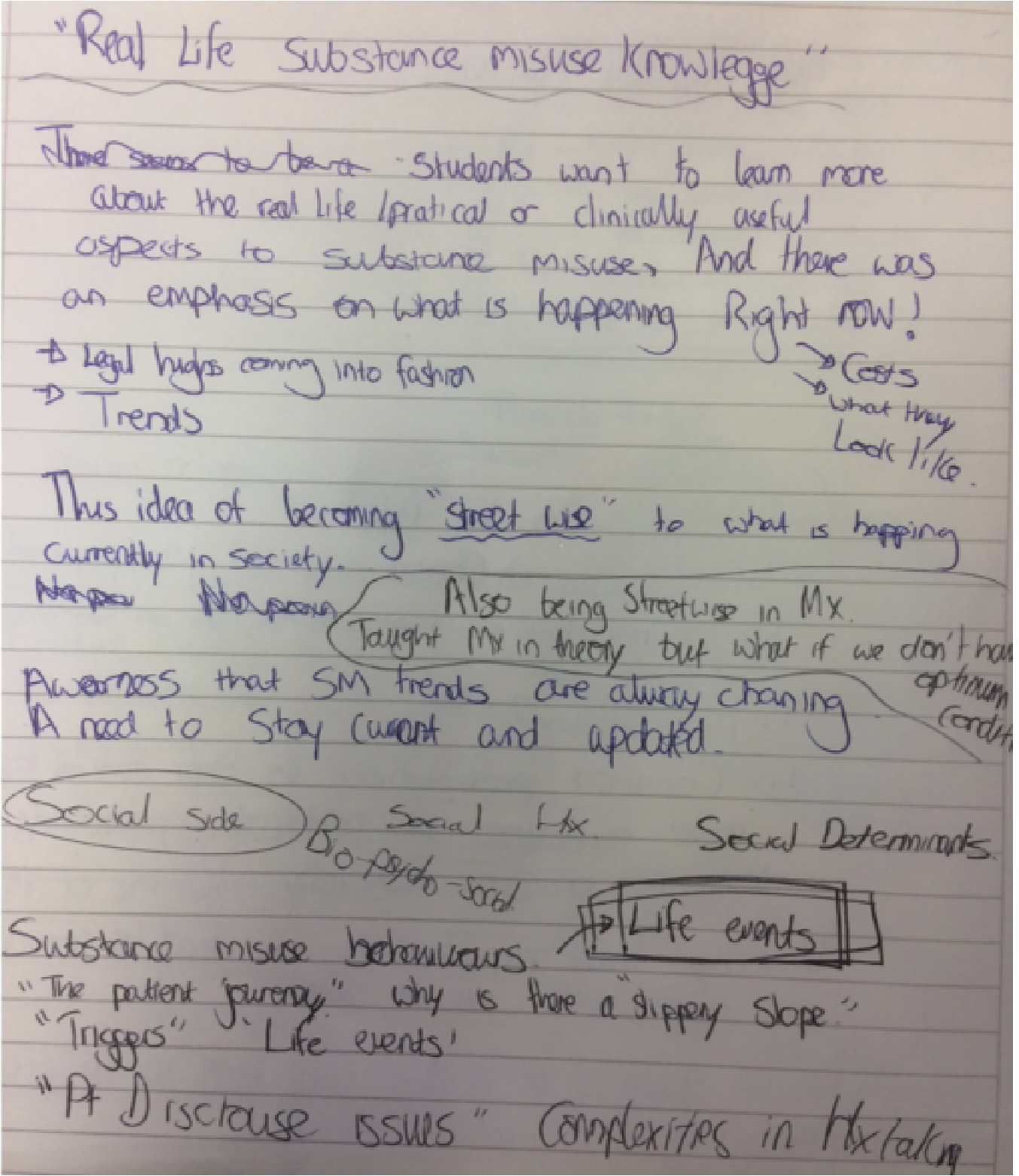


**Appendix 4 – Pre-conceived idea / thoughts memo**


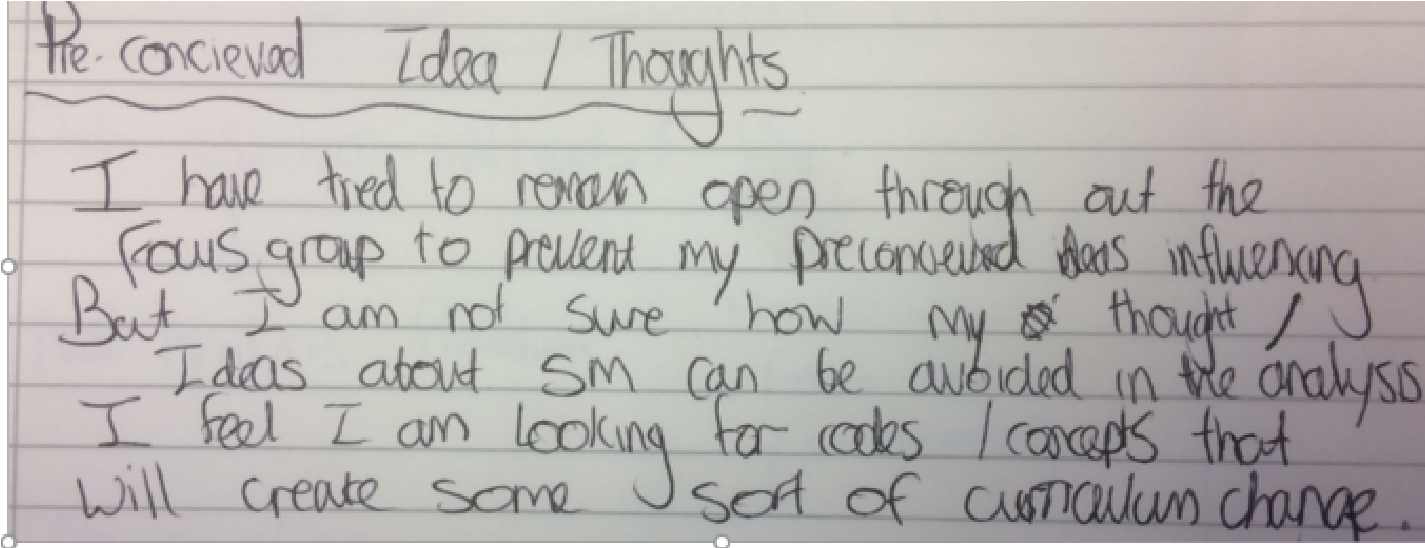


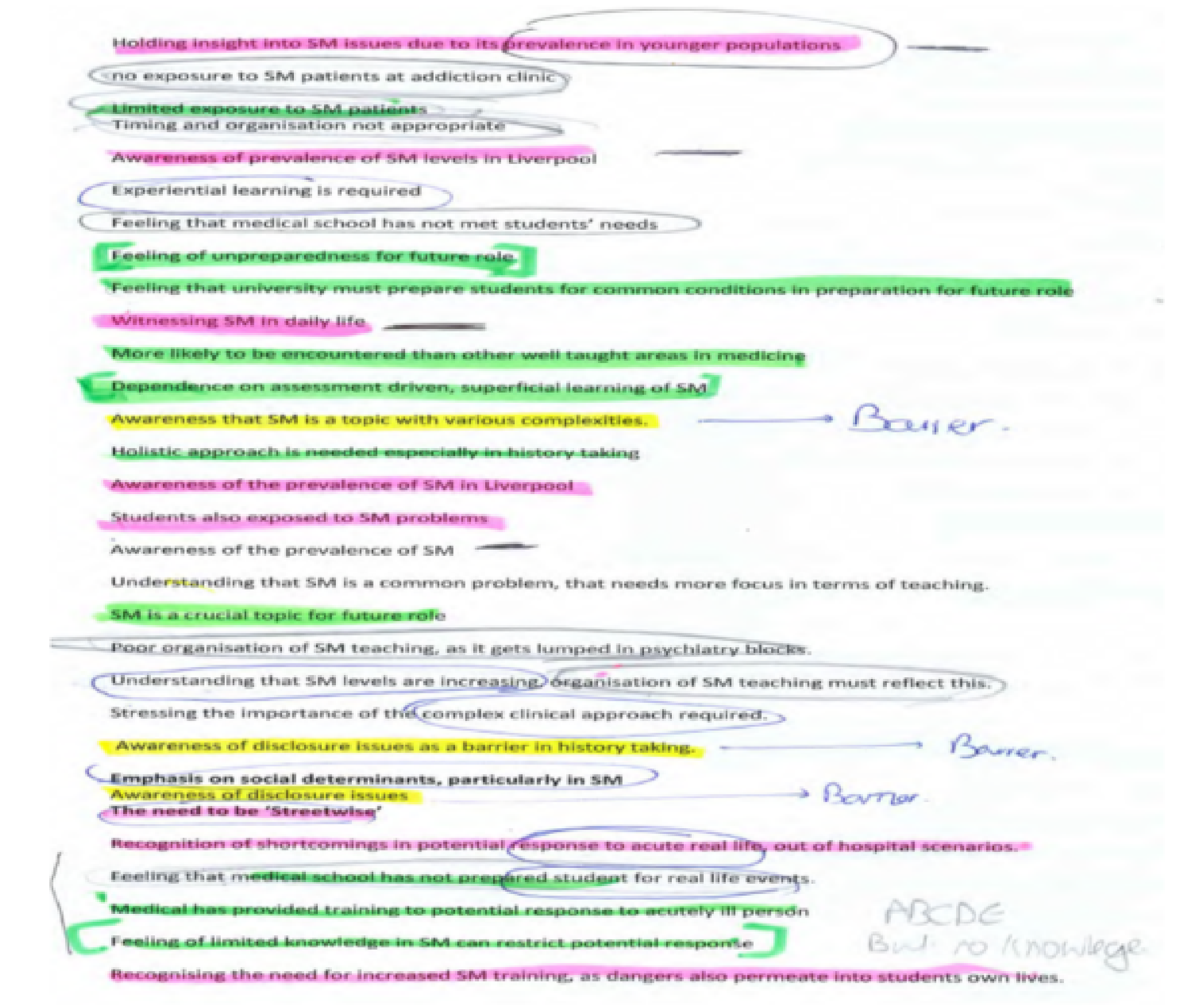

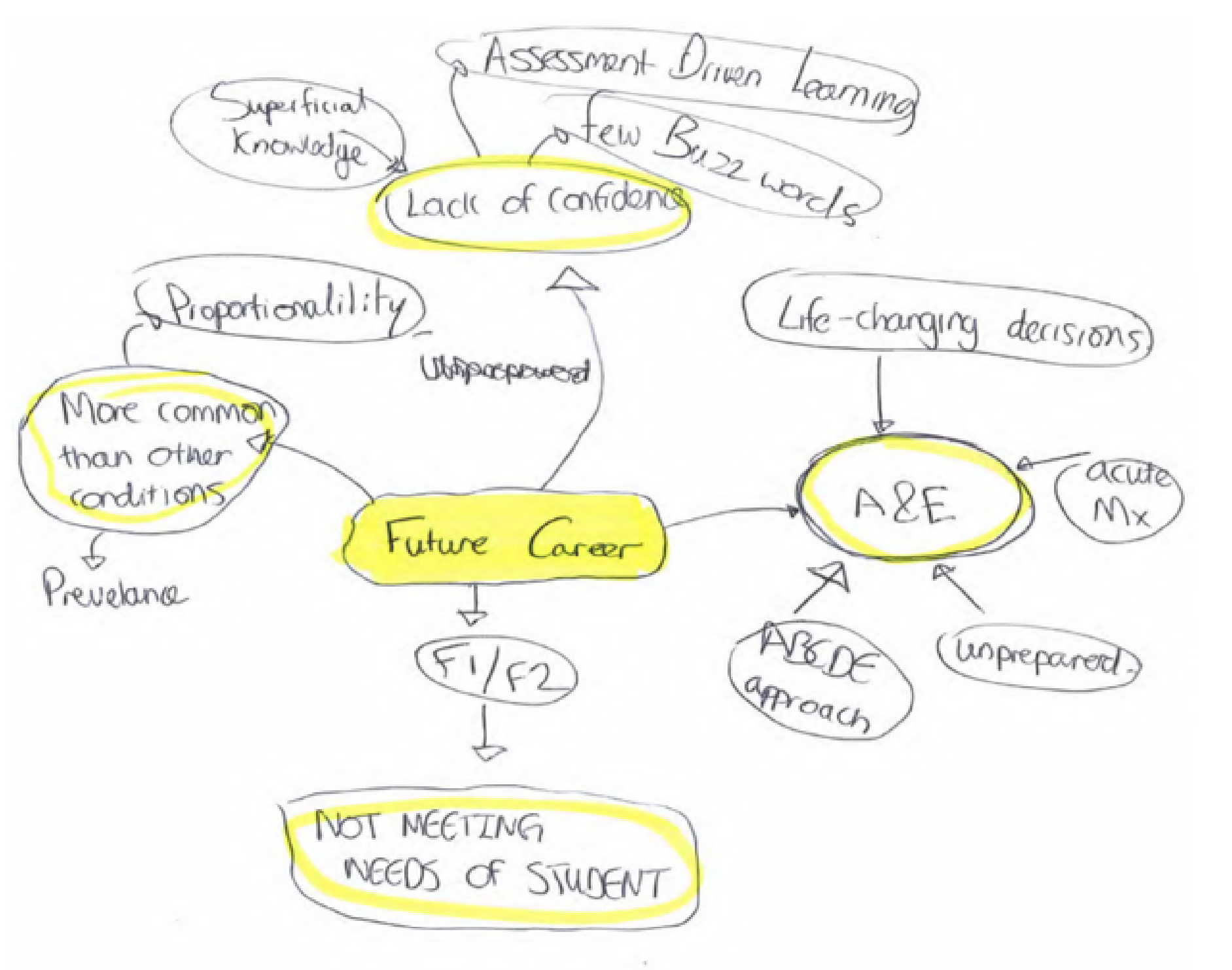
**Appendix 5 – Formation of future Careers cluster**
